# Evidence of a local negative role for cocaine and amphetamine regulated transcript (CART), inhibins and low molecular weight insulin like growth factor binding proteins in regulation of granulosa cell estradiol production during follicular waves in cattle

**DOI:** 10.1186/1477-7827-4-22

**Published:** 2006-04-12

**Authors:** Yasuhiro Kobayashi, Fermin Jimenez-Krassel, James J Ireland, George W Smith

**Affiliations:** 1 Department of Animal Science, Michigan State University, East Lansing, MI, USA; 2 Department of Physiology, Michigan State University, East Lansing, MI, USA

## Abstract

The ability of ovarian follicles to produce large amounts of estradiol is a hallmark of follicle health status. Estradiol producing capacity is lost in ovarian follicles before morphological signs of atresia. A prominent wave like pattern of growth of antral follicles is characteristic of monotocous species such as cattle, horses and humans. While our knowledge of the role of pituitary gonadotropins in support of antral follicle growth and development is well established, the intrinsic factors that suppress estradiol production and may help promote atresia during follicular waves are not well understood. Numerous growth factors and cytokines have been reported to suppress granulosa cell estradiol production in vitro, but the association of expression of many such factors in vivo with follicle health status and their physiological significance are not clear. The purpose of this review is to discuss the in vivo and in vitro evidence supporting a local physiological role for cocaine and amphetamine regulated transcript, inhibins and low molecular weight insulin like growth factor binding proteins in negative regulation of granulosa cell estradiol production, with emphasis on evidence from the bovine model system.

## Introduction

Folliculogenesis is generally defined as the formation of mature preovulatory follicles (Graafian) from the pool of primordial (non-growing) follicles. Primordial follicles continuously enter the growing pool, but greater than 99% of all growing follicles die by atresia. In a landmark paper, Hodgen (1982) initially coined the terms recruitment, selection and dominance to describe the process of folliculogenesis [1]. Since then, numerous studies using both histological approaches and real-time ultrasonography have led to further refinements in definitions of the various stages of folliculogenesis (reviewed in [2-4]). Ireland et al [2] defined recruitment as the process whereby a cohort of primordial follicles begins to grow and becomes dependent on pituitary gonadotropin support (at the antral follicle stage of development) in order to continue development towards ovulatory size. Selection is the process whereby some antral follicles avoid atresia and become competent to achieve a species-specific ovulation quota. The process of selection is completed when the number of growing follicles is reduced to the exact number of follicles that eventually undergo ovulation [2]. Dominance is the process whereby a single follicle achieves and maintains prominence over the other recruited (subordinate) follicles in the cohort. The dominant follicle that develops during the luteal phase eventually loses its capacity to produce estradiol and undergoes atresia. In contrast, the dominant follicle that develops during the follicular phase ovulates. Demise of the dominant follicle through atresia (non-ovulatory wave) or ovulation and subsequent development of a new dominant follicle are crucial for maintenance of normal reproductive cycles. While our knowledge of the role of pituitary gonadotropins in support of antral follicle growth and development is well established [3,5] , the intrinsic factors that help initiate and support growth or atresia of follicles at all stages of development are not well understood.

A prominent wave like pattern of growth of antral follicles is characteristic of monoovulatory species such as cattle, horses and humans [6-8]. Follicles are recruited continuously and develop to the antral stage without significant gonadotropin support [4]. However, the majority of antral follicles undergo atresia due to a lack of gonadotropin stimulation. A transient rise in serum FSH concentrations precedes initiation of each follicular wave and stimulates growth of multiple 2–4 mm antral follicles. Serum concentrations of FSH then reach their nadir at the time when a single follicle begins to outgrow the rest of the follicles in its cohort during each follicular wave [3,4,8,9]. This growing follicle is often called the dominant follicle and the time point when the dominant follicle begins to outgrow the rest of the cohort is called deviation. As follicles approach deviation, the future dominant follicle begins to produce significantly higher amounts of estradiol compared to the rest of the follicles within the cohort [10]. Remaining subordinate follicles in the cohort stop producing estradiol as their growth becomes arrested. Enhanced LH pulsatility associated with luteolysis during the follicular phase rescues the dominant follicle and facilitates a further increase in estradiol production culminating in initiation of the preovulatory LH surge. In the absence of luteolysis, dominant follicles of nonovulatory waves ultimately experience a reduction in capacity to produce estradiol and undergo atresia.

One of the typical characteristics of follicles undergoing atresia is loss of ability to produce estradiol [11] and a decrease in the intrafollicular ratio of estradiol to progesterone (E:P). Estrogen active follicles with high E:P have biochemical characteristics of healthy growing follicles and a lower incidence of atresia compared to estrogen inactive follicles with a low E: P [11-13]. Xu et al [14] collected dominant follicles on day 2, 4, 6, 8 or 10 after emergence of the first follicular wave in cattle. While follicular fluid estradiol concentrations were reduced by day 6 in this study, morphological signs of atresia were not readily detectable in granulosa cells of the dominant follicle until 10 days after wave emergence [14] , indicating that granulosa cells of dominant follicles lose their capacity to produce estradiol before undergoing atresia. In addition, Austin et al demonstrated that subordinate follicles also lose their capacity to produce estradiol before onset of atresia during follicular waves in cattle [15]. The ability of antral follicles to produce large amounts of estradiol is a cornerstone characteristic of follicle health status.

Along with gonadotropins, production of estradiol by granulosa cells is facilitated by complex interactions of many locally produced growth factors [16,17]. Paracrine actions of GDF-9 and BMP-15 (oocyte-derived members of the TGF-β superfamily) in regulation of granulosa cell estradiol production have received considerable attention due to genetic evidence for a requirement of such factors for normal follicular development (reviewed in [17-19]). Discussion of the potential role of such factors is regulation of granulosa cell steroidogenesis is beyond the scope of this review.

The insulin-like growth factors (IGF-I and -II) have also been implicated in regulation of granulosa cell estradiol production. The IGFs enhance FSH actions in vitro including stimulation of estradiol production [20,21] by granulosa cells of multiple species including cattle and increase FSH [22] and LH [23] receptor mRNA abundance in rat granulosa cells. In cattle, evidence indicates IGFs play an important regulatory role once follicles reach the antral stage and become gonadotropin dependent [24]. This further supports the notion that IGFs promote a synergistic enhancement of FSH action in antral follicles.

Granulosa cells also produce multiple members of the TGF-β superfamily including the inhibins and activins. The classical function of these two proteins is the modulation of pituitary FSH secretion [25,26]. Described local actions of activins in regulation of granulosa cell function included stimulation of increased rat granulosa cell LH and FSH receptors and enhancement or mediation of FSH stimulated estradiol production by granulosa cells of multiple species including cattle [27,28].

While exogenous FSH can rescue additional follicles in a cohort from atresia, the exact mechanisms involved in the reduction of estradiol production before atresia are unclear. Many factors, including cytokines (IL-1 alpha and beta, interleukin-2, interleukin-6 [29-37] and tumor necrosis factor-α [30,31,38] ); glucocorticoids [39-41], leptin [42-48], basic fibroblast growth factor [49] , epidermal growth factor [50,51] , TGF-α [51,52] and TGF-β [53,54] have been shown to inhibit basal, FSH and (or) growth factor stimulated estradiol biosynthesis in vitro in various species including cattle (Table 1). Nevertheless, evidence for regulation of such factors during antral follicle growth in vivo and the potential physiological contribution of many of these factors to regulation of estradiol production during follicular waves in cattle are not well understood. However, evidence obtained from multiple laboratories, including those of the authors, support a physiological role for the IGFBPs (reviewed in [55-57]) and inhibins [58] as negative regulators of granulosa cell estradiol production during follicular waves in cattle. Furthermore, the authors have recently obtained evidence of a potential physiological role for cocaine and amphetamine regulated transcript (CART) as a negative regulator of bovine granulosa cell estradiol production [59]. The purpose of the remainder of this minireview is to discuss the in vivo and in vitro evidence supporting a physiological role for low molecular weight IGFBPs, inhibins and CART in negative regulation of granulosa cell estradiol production during follicular waves in cattle. Our emphasis is on the bovine model, rather than comparative discussions of growth factor regulation of estradiol production in different species. Cattle are a useful model for studies of regulation of estradiol production because: 1) cattle [6] , like humans [8] , have distinct follicular waves which can be monitored by ultrasonography throughout the reproductive cycle, 2) monitoring of follicular waves by ultrasonography allows for collection of granulosa cells at specific defined stages of differentiation and 3) bovine granulosa cells harvested at specific stages of follicular development can be cultured under serum-free conditions that maintain a cellular phenotype similar to that of cells at time of collection [60] , thus facilitating investigation of the physiological role of local factors in control of estradiol production.

**Table I:** Factors that inhibit granulosa cell estradiol production.

Factor	Species	Reference	Comments
Tumor necrosis factor-α	Cows, rats	[30, 31, 38]	
Glucocorticoids	Cows, hamsters, pigs	[39-41]	
Leptin	cows, rats, humans, sheep	[42-48]	Passive immunization caused acute elevation in E production, whereas administration of low dose leptin into vena cava caused acute decrease in E production [48].
			
Basic fibroblast growth factor	Cows	[49]	
Transforming growth factor-α	Cows, mice, pigs	[51, 52]	
Transforming growth factor-β	Cows, mice, pigs	[53, 54]	
Epidermal growth factor	Sheep, mouse	[50, 51]	
Interleukin-1 (α and β)	Cows, rats, humans	[30-35]	Effects were mediated by nitric oxide [35].
Interleukin-2	Cows	[30]	
Interleukin-6	Cows, humans	[29,30,36,37]	
Interferon β	Cows	[30]	
Interferon γ	Cows	[30]	
Interferon τ	Cows	[30]	

### IGFBP regulation of granulosa cell estradiol production

During the past two decades, the role of IGFs and their binding proteins (IGFBP-1 through 6) in regulation of follicular growth and steroidogenesis have been studied extensively (reviewed in [20,21,24,56,61]. IGF bioavailability is regulated by local concentrations of the low molecular weight IGF binding proteins (IGFBPs), which thus play a negative role in regulation of follicular estradiol production. IGF binding proteins-2, -3, -4, and -5 are detectable in the follicular fluid of cattle [20,21]. The relative amounts of different IGFBPs change during follicular development and atresia [20,21,62,63]. Amounts of IGFBFP-2 [64-66], IGFBP-4 [10,64,67] and IGFBP-5 [64,68] are greater in subordinate follicles than in the dominant follicle, whereas amounts of IGFBP-3 are not different [64,68]. Concentrations of free IGF-I in follicular fluid are greater in the dominant follicle compared to subordinate follicles [65,69] , but concentrations of free IGF-I in follicular fluid of the dominant follicle do not change during it's growth.

Available evidence indicates that amounts of IGFBPs within follicular fluid are regulated by multiple factors including gonadotropins, proteolytic enzymes, and extracellular matrix proteins [61,70]. In particular, proteolysis is an important mechanism for regulation of IGFBP-2, IGFBP-4 and IGFBP-5 concentrations in follicular fluid. Proteolytic activity degrading IGFBP-4 [67,71-73] and IGFBP-5 [72,73] was greater in the dominant follicle compared to subordinate follicles in cattle and bovine preovulatory follicular fluid was shown to degrade IGFBP-2 [74]. One of the major proteolytic enzymes involved in IGFBP degradation is pregnancy-associated plasma protein A (PAPP-A), a zinc-dependent metalloproteinase [75]. PAPP-A is present in bovine follicular fluid [73,76]. Amounts of PAPP-A mRNA are higher in dominant versus subordinate follicles [77]. Studies by different laboratories [67,71-73,76] demonstrated that amounts of IGFBP-4 in follicular fluid are regulated by proteolysis. Rivera and Fortune [72,73] further demonstrated that IGFBP-5 in follicular fluid also is susceptible to proteolysis. Treatment of recombinant IGFBP-4 and IGFBP-5 with bovine follicular fluid collected from the preovulatory follicle (bFF) increased proteolysis of IGFBP-4 and IGFBP-5, and proteolysis of both binding proteins was reduced following immunoneutralization and immunoprecipitation of PAPP-A from the follicular fluid [73].

Given the previously described direct and indirect actions of IGF-I that promote estradiol production, evidence supports a key role for the IGFBPs in regulation of IGF-I bioavailability and thus a potentially important indirect role in inhibition of estradiol biosynthesis during the early stages of a follicular wave. In fact, differences in the amount of follicular fluid IGFBP-4 between the future dominant and subordinate follicles are detectable prior to deviation and associated with steroidogenic capacity. Mihm et al [10] removed small amounts of follicular fluid from the three largest follicles on day 1.5 after emergence of the first follicular wave (prior to deviation). The fate of each follicle was monitored ultrasonographically after sample collection. Prior to deviation, the follicles that eventually became dominant had greater estradiol concentrations compared to those follicles that eventually became the subordinate follicles (Table 2). In comparison, amounts of IGFBP-4 were less in the future dominant versus subordinate follicles (Table 2). The results of this study are important because all follicles were estrogen active at the time of collection, suggesting that changes in IGF-I bioavailability occur before subordinate follicles lose their capacity to produce estradiol.

**Table II:** IGFBPs and estradiol in follicular fluid from future dominant and subordinate follicles prior to deviation.

Parameters	Future dominant follicle	Future subordinate follicle #1	Future subordinate follicle #2
Diameter (mm)	7.6 ± 0.4	6.9 ± 0.4	6.1 ± 0.4*
Follicular fluid estradiol (E; ng/ml)	113 ± 30	44 ± 11*	40 ± 13*
Follicular fluid progesterone (P; ng/ml)	21 ± 3	42 ± 27	14 ± 3
E:P ratio	5.2 ± 0.8	3.1 ± 0.8*	2.7 ± 0.4*
IGFBP-2 (Arbitrary units)	12 ± 2	18 ± 4	14 ± 5
IGFBP-3 (Arbitrary units)	203 ± 17	245 ± 28	197 ± 36
IGFBP-4 (Arbitrary units)	1 ± 0.3	14 ± 7*	11 ± 4*
IGFBP-5 (Arbitrary units)	13 ± 2	32 ± 10	24 ± 8

* Significantly different (P < 0.05) from the future dominant follicle (Adapted from Mihm et al., 2000 [10])

Collectively, results suggest that increased expression of IGFBP degrading proteinases, such as PAPP-A may help account for described differences in IGFBPs in dominant versus subordinate follicles in cattle and preferentially protect the dominant follicle from the negative effects of IGFBP-4 and IGFBP-5, resulting in enhanced IGF-I bioavailability and estradiol production. Whether proteolytic degradation is the mechanism responsible for reduced amounts of IGFBP-4 observed in the future dominant versus subordinate follicles collected prior to deviation remains to be determined. Furthermore, complementary direct in vitro evidence for a negative effect of exogenous IGFBPs on estradiol production is limited. Spicer and Chamberlain observed an inhibitory effect of recombinant human IGFBP3 on IGF-I stimulated bovine granulosa cell estradiol production, but no effect on FSH stimulated estradiol production [78] was noted. IGFBP-4 does inhibit FSH or LH stimulated estradiol production by human granulosa cells [79]. However, greater biopotency of IGF-1 analogs in vitro (such as LR3-IGF-1 that do not bind well to IGFBPs, but have similar or slightly reduced receptor binding affinity) versus IGF-I in stimulating estradiol production provides indirect evidence for a negative effect of endogenous IGFBPs on bovine granulosa cell estradiol production [80]. Investigation of effects of various IGFBPs, alone or in combination, on IGF action during folliculogenesis will help further clarify their physiological role in regulation of estradiol production.

### Regulation of granulosa cell estradiol production by inhibins

The inhibins are isomeric glycosylated proteins produced by granulosa cells, with inhibin A composed of the inhibin α subunit and β A subunit and inhibin B containing the β B subunit in place of the β A subunit [27]. Granulosa cells also produce activins; activin A (composed of two β A subunits), activin AB (composed of β A and β B subunit), and activin B (composed of two β B subunits) [25-27]. The activins and inhibins were first characterized as proteins present in follicular fluid that stimulate or suppress pituitary FSH secretion respectively [25,26,81] , but now are also known for their pleiotropic actions characteristic of members of the TGF-β growth factor superfamily [82]. The role of activins and inhibins in regulation of follicular growth and (or) steroidogenesis has been addressed in detail in several previous reviews [5,17,25].

Actions of activins and inhibins are mediated through interactions with specific membrane receptors of the serine threonine kinase subtype [25]. One activin molecule binds to two activin receptors (type I and type II), resulting in activation of the SMAD second messenger system [83]. Activated SMADS then enter the nucleus and activate gene transcription [83]. Evidence to date suggests that biological actions of inhibins are mediated through inhibition of activin action occurring primarily via antagonism of activin binding to its receptors [83,84]. The type II activin receptor has a very low affinity for inhibins [25,83,85,86] and thus inhibin does not efficiently compete directly for activin binding. Lewis et al [84] demonstrated that antagonism of activin action by inhibins requires interaction between inhibins and betaglycan (the type III TGF-β receptor). Binding of inhibin to the inhibin co-receptor betaglycan increases affinity of inhibin for type II activin receptors [25,83,85,86]. Gray et al [86] suggested that formation of inhibin-betaglycan-type II activin receptor ternary complex prevents binding of activin to type II receptors, which subsequently inhibits activin actions. Whether formation of the inhibin-betaglycan-type II activin receptor ternary complex can also activate intracellular signaling pathways remains unclear because betaglycan has a very short cytoplasmic tail lacking intrinsic signaling domains [84,86].

Evidence supports an intrafollicular regulatory role for activin in stimulation of granulosa cell estradiol production. Activin treatment of bovine granulosa cells results in enhanced aromatase activity and estradiol production and a suppression of granulosa cell progesterone production [87,88]. Furthermore, activins may help mediate the stimulatory effects of FSH on granulosa cell estradiol production. FSH stimulates granulosa cell activin production and addition of the activin binding protein follistatin results in a reduction in FSH stimulated estradiol production by bovine granulosa cells [28].

As opposed to the stimulatory effects of activins, direct and indirect evidence supports an inhibitory role for the inhibins in regulation of bovine granulosa cell estradiol production. Amounts of inhibins in the follicular fluid and inhibin α subunit mRNA in granulosa cells are greater in healthy follicles compared to atretic follicles at several stages of development [89-92]. Furthermore, a pronounced negative relationship between follicular fluid concentrations of inhibin A and estradiol occurs throughout dominant follicle development in cattle in the absence of changes in intrafollicular activin A and follistatin [93-96]. In contrast, Bleach et al [97] showed that circulating inhibin A concentrations were positively correlated with concentrations of estradiol and LH, whereas circulating concentrations of FSH were negatively correlated with inhibin A concentrations. Studies using nonluteinized cultured granulosa cells showed that in vitro production of inhibin A was stimulated by FSH in sheep [91] and cattle [28]. Plasma concentrations of inhibin A decreased once the dominant follicle lost its ability to produce estradiol. The difference in plasma versus follicular fluid inhibin A concentrations was potentially attributed to loss of blood vessels in the dominant follicle during atresia [97]. However, the exact mechanisms responsible for such maintenance of elevated inhibin A concentrations in follicular fluid are unclear. Whether inhibins act solely as an antagonist of activin action on granulosa cells or they also affect granulosa cell function through novel pathways is not known. Messenger RNA for the inhibin co-receptor betaglycan is greater in granulosa cells of subordinate versus dominant follicles collected near the time of deviation (3 days after emergence) of the first follicular wave in cattle [98] , suggestive of a potential role for betaglycan regulation in control of inhibin mediated antagonism of activin action resulting in reduced estradiol production in subordinate follicles.

**Figure 1:**
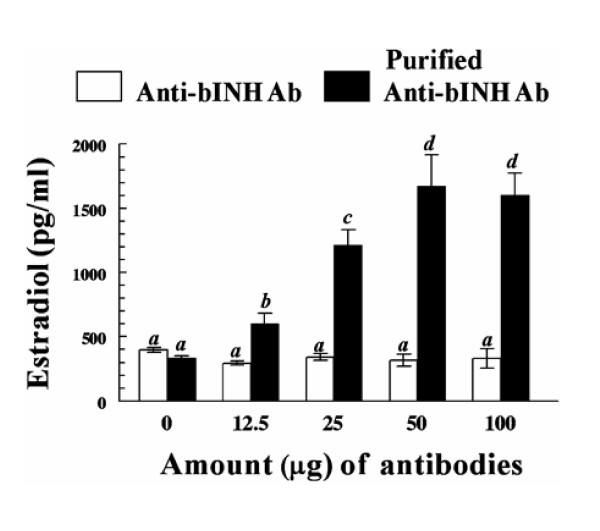
Dose-response effect of purified bovine anti-bovine inhibin antibodies on bovine granulosa cell estradiol production. Estrogen-inactive dominant nonovulatory follicles were obtained from a local abattoir. Granulosa cells from each follicle were pooled and treated for 18 hours with different doses of purified bovine anti-bovine inhibin antibodies (purified anti-bINH Ab) or partially purified bovine anti-bovine inhibin antibodies (anti-bINH Ab). Bars represent the mean ± SEM values for triplicate determinations of concentrations of estradiol in media after culture of a single pool of granulosa cells isolated from two follicles (n = 2 cows). Different letters above bars indicate significant (*P *< 0.05) difference in means. Reproduced from Jimenez-Krassel et al., 2003 [58].

**Figure 2:**
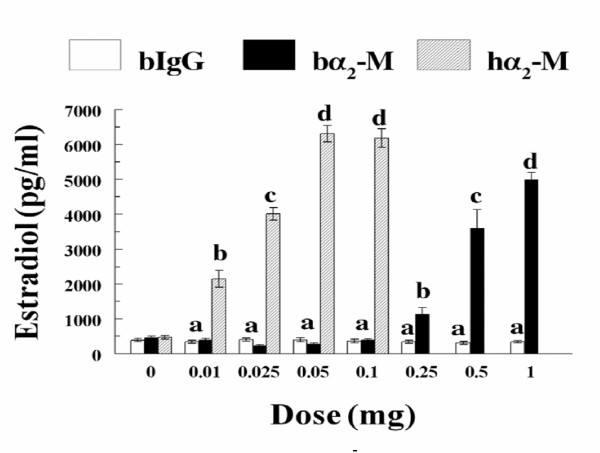
Dose response effect of α_2_-macroglobulin on bovine granulosa cell estradiol production. Granulosa cells from dominant nonovulatory follicles were treated for 18 hours with different amounts of bovine IgG (control), bovine α_2_-macroglobulin (bα_2_-m) or human α_2_-macroglobulin (hα_2_-m). Bars represent the overall mean ± SEM values for concentrations of estradiol in media after culture of granulosa cells isolated from two follicles (n = 2 cows). Different letters above bars indicate significant (*P *< 0.01) difference among means. Reproduced from Ireland et al., 2004 [101].

Above indirect evidence is supportive of a potential negative role for inhibins in regulation of granulosa cell estradiol production in cattle. However, studies examining direct effects of inhibins on granulosa cell estradiol production in vitro in other species, including sheep [91] , rodents [87,99] , and primates [100] have yielded conflicting results. However, Jimenez-Krassel et al [58] recently reported direct evidence for a potent negative effect of inhibins on bovine granulosa cell estradiol production in vitro. Using a short term, serum free culture system where cells maintain a state of differentiation reflective of their steroidogenic capacity at time of collection [60] , immunoneutralization of endogenous inhibin with bovine anti-bovine inhibin antibodies resulted in pronounced dose dependent increases in capacity of cells to produce estradiol (Figure 1), regardless of whether cells were collected from estrogen active or inactive dominant nonovulatory follicles, dominant ovulatory follicles or subordinate follicles [58]. Furthermore, a dose dependent decrease in estradiol production was observed when bovine granulosa cells were incubated with increasing concentrations of immunoaffinity purified bovine inhibins [58]. In addition to the inhibin co-receptor betaglycan, the proteinase inhibitor α_2 _-macroglobulin, a proteinase inhibitor in high concentrations in follicular fluid that also binds inhibins, may have an important role in regulation of negative action of inhibins on bovine granulosa cell estradiol production. Recent studies [101] using short-term cultures demonstrated that treatment of bovine granulosa cells from dominant and subordinate follicles with α_2_-macroglobulin robustly enhanced estradiol production in a dose response fashion (Figure 2). However, it is unknown whether the positive effect of α_2_-macroglobulin is mediated indirectly by binding of inhibins to α_2_-macroglobulin thus preventing interaction of inhibin with betaglycan, or directly via binding of the inhibin- α_2_-macroglobulin complex to the α_2_-macroglobulin receptor.

Collectively, above described negative relationship between follicular fluid estradiol and inhibin concentrations, enhanced expression of betaglycan mRNA in granulosa cells of subordinate versus dominant follicles, and direct effects of α_2_-macroglobulin (the inhibin binding protein) and inhibins on bovine granulosa cell estradiol production observed in vitro are supportive of a potential local physiological role for inhibins in negative regulation of granulosa cell estradiol production during follicular waves in cattle. Whether the negative effects of inhibin on granulosa cell estradiol production are mediated solely via interaction with betaglycan and subsequent antagonism of activin action, indirectly via α_2_-macroglobulin, or via activation of novel pathways, remains to be determined.

### Regulation of granulosa cell estradiol production by cocaine and amphetamine regulated transcript (CART)

The advent of functional genomics has sparked new opportunities to discover and investigate novel intrafollicular regulatory molecules that modulate granulosa cell function. During experiments to characterize the transcriptome of bovine oocytes and generate an oocyte cDNA microarray for physiological studies, five expressed sequence tags (ESTs) with similarity to a neuropeptide known as cocaine and amphetamine regulated transcript (CART) were identified [102]. Expression of CART mRNA in the mammalian ovary had not been reported previously. CART was initially discovered during differential display RT-PCR experiments focused on identification of transcripts induced in the brain of rats after administration of cocaine and amphetamine [103]. Since its discovery, reports of CART expression have been restricted primarily to neural tissues [104,105]. CART is particularly abundant in the hypothalamus of both humans and rodents [106-111] and evidence supports an important role for hypothalamic CART in regulation of feeding behavior [109,112,113]. Within the ovary, CART mRNA (cell type of origin unknown) has been detected in goldfish [114]. In the male gonad, CART expression has been observed in neurons innervating the epididymis of rats [115].

After initial sequencing of CART ESTs from the bovine oocyte cDNA library, expression of CART in additional ovarian cell types was investigated. In situ hybridization, immunohistochemistry and RT-PCR experiments confirmed oocyte expression of CART, but also revealed prominent localization of CART mRNA and the CART peptide in the granulosa layer of some, but not all antral follicles examined [59]. Expression of CART was not detected in preantral follicles. Given the robust expression of CART observed in granulosa cells versus the oocyte and observed localization of CART in some, but not all antral follicles [59] , subsequent experiments focused on the regulation and regulatory role of granulosa cell derived CART during follicular waves in cattle.

**Figure 3:**
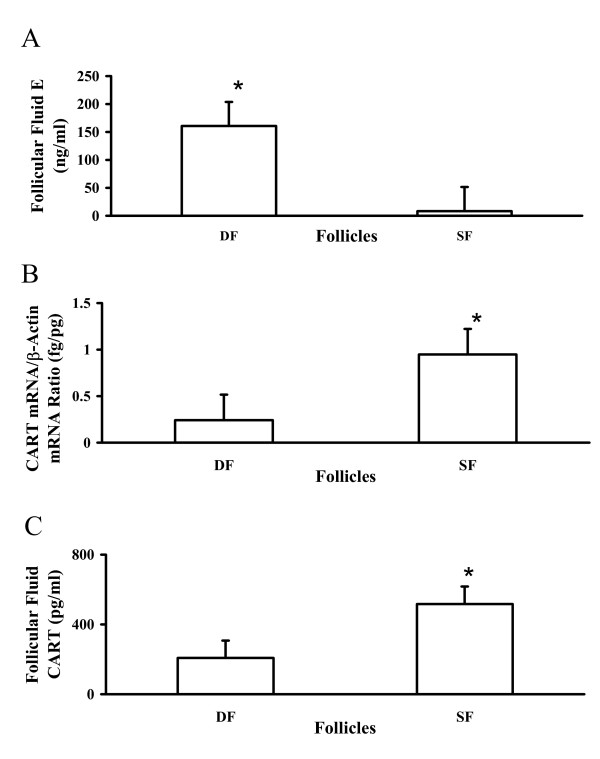
Expression of CART mRNA and follicular fluid concentrations of CART peptide in healthy estrogen-active dominant follicles (DF) and atretic estrogen-inactive subordinate follicles (SF) collected at random stages of the first follicular wave. A) Intrafollicular concentrations of estradiol (E) in estrogen-active DF and estrogen-inactive SF collected at random stages of the first follicular wave (n = 5 each; least square means +/- SEM; * P < 0.05 versus SF). B) Relative abundance of CART mRNA in estrogen-active DF and estrogen-inactive SF collected at random stages of the first follicular wave (n = 5 each). Expression of CART mRNA was normalized relative to concentrations of β-actin mRNA and expressed as ratio of femtograms CART mRNA to picograms β-actin mRNA (least square means +/- SEM). Expression of CART mRNA was higher in estrogen-inactive SF relative to healthy estrogen-active DF (* P < 0.05). C) Intrafollicular concentrations of CART peptide in estrogen-active DF and estrogen-inactive SF collected at random stages of the first follicular wave (n = 4 each; least square means +/- SEM). Concentrations of CART peptide were also greater in follicular fluid of estrogen-inactive SF relative to estrogen-active DF (* P < 0.05). Reproduced from Kobayashi et al., 2004 [59].

**Figure 4:**
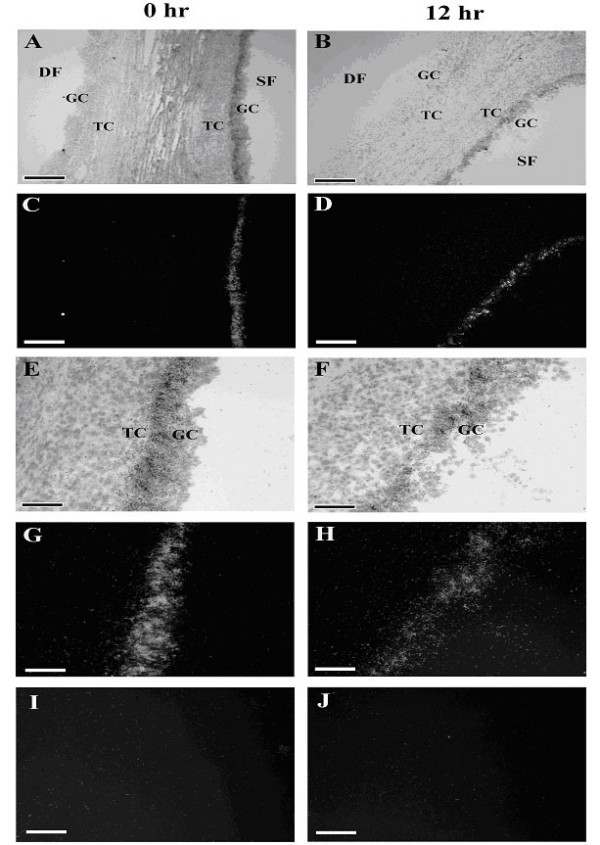
In situ localization of CART mRNA within granulosa cells of dominant ovulatory versus nonovulatory (subordinate) bovine follicles collected before (0 h) and after (12 h) GnRH injection to induce the preovulatory gonadotropin surge (n = 3 each). A) Bright field micrograph of section through dominant ovulatory follicle (denoted as DF) and adjacent non-ovulatory subordinate follicle (denoted as SF) collected before the preovulatory gonadotropin surge (0 h timepoint) and hybridized with ^35^S-antisense CART cRNA. B) Bright field micrograph of section through DF and adjacent SF collected after the preovulatory gonadotropin surge (12 h timepoint) and hybridized with ^35^S-antisense CART cRNA. C) Dark field micrograph of section adjacent to one depicted in Panel A. D) Dark field micrograph of section adjacent to one depicted in Panel B. Note localization of CART mRNA to GC of the SF (right) but not the DF (left) depicted in Panels A-D. E) Magnified bright field micrograph of SF depicted in Panels A and C. F) Magnified bright field micrograph of SF depicted in Panels B and D. G) Magnified dark field micrograph of Panel E. H) Magnified dark field micrograph of Panel F. Note localization of CART mRNA specifically to GC of SF in panels G and H. I and J) Dark field micrographs of sections adjacent to ones depicted in Panels E and F hybridized with ^35^S-sense CART cRNA (negative control). A-D: magnification = 100X, scale bar = 200 μm; E-J: magnification = 400X, scale bar = 50 μm. Granulosa cell layer denoted as GC. Theca cell layer denoted as TC. Reproduced from Kobayashi et al., 2004 [59].

Available evidence supports an inhibitory role of CART in regulation of granulosa cell estradiol production. A negative relationship between CART expression and follicle health status was observed. As illustrated in Figure 3, CART mRNA and follicular fluid concentrations of the CART peptide were greater in estrogen inactive subordinate follicles versus estrogen active dominant follicles collected at random stages of the first follicular wave [59]. The association between follicle health status and CART expression is also illustrated in Figure 4. Note undetectable CART expression in the healthy dominant ovulatory follicles (DF; found on the left in both columns) collected at 0 and 12 h after GnRH injection to induce the preovulatory LH surge versus the prominent CART expression detected in the granulosa layer of the adjacent subordinate follicles (SF; found on the right in both columns). Given the observed negative association of CART expression with follicle health status, direct effects of CART treatment on granulosa cell estradiol production were then examined. Using the same short term granulosa cell culture system as described above [60] , bovine granulosa cells from dominant follicles collected at random stages of the first follicular wave were treated for 18 h with increasing concentrations of the CART peptide. Treatment with CART decreased bovine granulosa cell estradiol production in a dose dependent manner (maximal inhibition ~ 30%) by cells with high estradiol producing capacity in vivo at time of collection (> 50 ng/ml estradiol in follicular fluid) and in vitro (> 400 pg/ml estradiol), but not by cells with low estradiol producing capacity in vivo at time of collection (< 5 ng/ml estradiol in follicular fluid) and in vitro (< 400 pg/ml estradiol) [59]. Collectively, results support a potential physiological role for CART in regulation of bovine granulosa cell estradiol production. Effects of CART on FSH and growth factor (IGF-1) stimulated estradiol production by bovine granulosa cells and the mechanism of action of CART are currently being investigated.

The intracellular signaling pathways that mediate the negative effects of CART on granulosa cell estradiol production are currently unknown. Receptors for the CART peptide have not been cloned to date. However, in neurons, CART can inhibit L-type calcium channel activity [116] and FSH induced increases in granulosa cell calcium uptake are mediated via inhibition of L-type calcium channel activity [117]. Whether the negative effects of CART on bovine granulosa cell estradiol production are mediated via inhibition of L-type calcium channel activity or are calcium independent and mediated via a putative CART receptor is a subject for future investigation, as are the factors that regulate granulosa cell CART expression during follicular waves.

## Conclusion

From a physiological viewpoint, atresia of dominant follicles during non-ovulatory follicular waves is crucial for the timely development of ovulatory waves during the follicular phase of menstrual and estrous cycles in mono-ovulatory species like humans and cattle. Moreover, development of a single dominant ovulatory follicle from the cohort of growing follicles depends on the timely atresia of all subordinate follicles during waves. Consequently, it is crucial to understand the mechanisms that promote a reduction in estradiol production and onset of atresia of follicles during follicular waves. Morphological signs of atresia are preceded by decreased production of estradiol in the granulosa cells of antral follicles [11-15]. While the important endocrine role of pituitary gonadotropins in promoting granulosa cell estradiol production during follicular waves is well established, a physiological role for local regulatory factors in suppression of estradiol production prior to atresia cannot be discounted. The mechanism of action of IGFBP-4 and IGFBP-5 in regulation of estradiol production through modulation of intrafollicular IGF bioavailability has been well defined. However, the mechanism of action of inhibins and CART in regulation of granulosa cell estradiol production is unclear, but negative effects on basal and gonadotropin stimulated aromatase mRNA expression and enzyme activity seem plausible. Furthermore, local effects of IGFBPs, inhibins and CART on granulosa cell estradiol production in vivo during follicular waves in cattle have not been directly tested, but can potentially be explored using ultrasound mediated intrafollicular injection procedures [118]. Further examination of the mechanism of action of above factors (inhibins and CART) and identification of additional local regulators of granulosa cell steroidogenesis will facilitate the discovery of additional pathways contributing to decreased estradiol production and eventual demise of ovarian follicles.
